# Pseudo-Chromosomal Genome Assembly in Combination with Comprehensive Transcriptome Analysis in *Agaricus bisporus* Strain KMCC00540 Reveals Mechanical Stimulus Responsive Genes Associated with Browning Effect

**DOI:** 10.3390/jof8080886

**Published:** 2022-08-22

**Authors:** Ick-Hyun Jo, Jaewook Kim, Hyejin An, Hwa-Yong Lee, Yoon-Sup So, Hojin Ryu, Gi-Ho Sung, Donghwan Shim, Jong-Wook Chung

**Affiliations:** 1 National Institute of Horticultural and Herbal Science, Rural Development Administration, Eumseong 27709, Korea; 2 Department of Biological Science, Chungnam National University, Daejeon 34134, Korea; 3 Department of Industrial Plant Science and Technology, Chungbuk National University, Cheongju 28644, Korea; 4 Department of Forest Science, Chungbuk National University, Cheongju 28644, Korea; 5 Department of Crop Science, Chungbuk National University, Cheongju 28644, Korea; 6 Department of Biology, Chungbuk National University, Cheongju 28644, Korea; 7 Institute for Bio-Medical Convergence, International St. Mary’s Hospital, College of Medicine Catholic Kwandong University, Incheon 21431, Korea

**Keywords:** *Agaricus bisporus*, de novo assembly, transcriptome analysis, mechanical stress, melanin

## Abstract

*Agaricus bisporus* is one of the world’s most popular edible mushrooms, including in South Korea. We performed de novo genome assembly with a South Korean white-colored cultivar of *A. bisporus*, KMCC00540. After generating a scaffold-level genomic sequence, we inferred chromosome-level assembly by genomic synteny analysis with the representative *A. bisporus* strains H97 and H39. The KMCC00540 genome had 13 pseudochromosomes comprising 33,030,236 bp mostly covering both strains. A comparative genomic analysis with cultivar H97 indicated that most genomic regions and annotated proteins were shared (over 90%), ensuring that our cultivar could be used as a representative genome. However, *A. bisporus* suffers from browning even from only a slight mechanical stimulus during transportation, which significantly lowers its commercial value. To identify which genes respond to a mechanical stimulus that induces browning, we performed a time-course transcriptome analysis based on the de novo assembled genome. Mechanical stimulus induces up-regulation in long fatty acid ligase activity-related genes, as well as melanin biosynthesis genes, especially at early time points. In summary, we assembled the chromosome-level genomic information on a Korean strain of *A. bisporus* and identified which genes respond to a mechanical stimulus, which provided key hints for improving the post-harvest biological control of *A. bisporus*.

## 1. Introduction

The button mushroom (*Agaricus bisporus*) belongs to the *Basidiomycota* division, and it is one of the world’s most popular edible mushrooms, including in South Korea. *A. bisporus* primarily contains dietary fiber and varieties of polysaccharides, β-glucans, homoglucans, and heteroglycans, which are expected to have antioxidant and anticancer properties [1]. *A. bisporus* has variable cultivars that are white or brownish strains [2]. 

However, commercially well-accepted varieties are white strains that readily brown [3]. Thus, one major goal of *A. bisporus* distribution in food chains was to inhibit the browning phenotype [4,5,6,7].

Many high-quality genomes were assembled, especially at the chromosome level, for few strains [8,9,10]. However, most genomic information was assembled with U.S. or European cultivars, and only one cultivar from Asia (*A. bisporus* strain AB58 from the Tibetan plateau) was available [8,9,10]. Although strain AB58 is Asian, the geographical distance of AB58 is large enough to expect major genomic variation with South Korean cultivars. South Korean scientists made an effort to breed *A. bisporus* strains using their own original strains [11]. White strain KMCC00540 originates from Daegu-shi is easy to grow and has superior morphology [12]. Due to its geographical localization, it was uncertain that it could be representative genetic material of *A. bisporus*.

The browning of white *A. bisporus* can be induced by several e including abiotic stimulus and biotic stress. The most well-known abiotic stimulus is mechanical stress such as simple shearing stress [13,14,15]. It has been suggested that mechanical stress/stimulus induces enzymatic reactions, including oxidative reactions. Thus, studies were mainly designed to restrict those reactions by covering the surface of *A. bisporus* or treating with simple chemicals that might interrupt the enzymes [4,5,6,7,16]. Meanwhile, biotic stress has also been identified as a cause of browning. Mushroom virus X had the potential to induce browning in *A. bisporus,* and several correlated genes were identified [17,18]. In a recent report, the rare bacterium *Cedecea neteri* also seemed to induce browning in *A. bisporus*, but this requires further study [19]. Well-known pathogenic inducers of browning includes cobweb disease from Cladobotryum spp. and wet bubble disease from Mycogone perniciosa [20].

Though the reasons for the browning of white *A. bisporus* have been studied in several aspects, little is known about how it is regulated. The most famous theory on the browning mechanism in *A. bisporus* is a polyphenol oxidase (PPO) enzyme-related pathway [21]. However, catalase (CAT) and superoxide dismutase (SOD) activity-related mechanisms have also been proposed [21]. Since CAT and SOD correlate with reactive oxygen species (ROS), lowering their activity was proposed as a mechanism to inhibit browning [21]. PPOs catalyze later steps of melanin biosynthesis, which creates brown/black pigments. Thus, PPO activity demonstrates that the melanin biosynthesis pathway is one of major mechanism of browning [22].

The melanin biosynthesis pathway has been proposed to produce four subtypes. When chorismate is catalyzed by 4-Aminobenzoate synthase (4ABS), it catalyzes into γ-l-glutaminyl-4-hydroxybenzene (GHB)-type melanin or *p*-aminophenol (PAP)-type melanin. On the other hand, if chorismate is catalyzed into prephenate by chorismate mutase (CM), it further catalyzes into l-3,4-dihydroxyphenylalanine (L-DOPA)-melanin or catechol-melanin [22]. Melanin pigment has yellow, reddish, and brown subtypes. For example, the L-DOPA-melanin type shows a canonical dark-brown or black color, while 5-cys-dopa-melanin is yellowish to reddish [23].

Through previous knowledge, we identified clear transcriptomic analyses of mechanical stimulus-induced changes in *A. bisporus* that will be required for improving our understanding of how browning occurs. Metabolomic analysis combined with transcriptomic analysis were performed together in easily browning *A. bisporus* cultivars and hardly browning cultivars. There was no clear correlation between the transcriptome and metabolome, but melanin biosynthesis genes were up-regulated in easily browning cultivars [24]. To study the effect of a mechanical stimulus on *A. bisporus* including browning, we used our own material, which also showed a browning phenotype after a mechanical stimulus.

We identified the genome of our cultivar as a standard genome of *A. bisporus* through the de novo assembly of the strain KMCC00540. Through comparative genomics analysis, we could identify that *A. bisporus* strain KMCC00540 shares most genomic blocks and similar genomic structures with the European strains H97 and H39. Thus, we confirmed that strain KMCC00540 could be used as a representative cultivar in a study of *A. bisporus*. To identify transcriptomic impacts of mechanical stimulus in *A. bisporus*, we applied a simple mechanical stimulus and performed transcriptomic analysis in a time-course manner. We identified many differentially expressed genes (DEGs), and among those, many gene ontology (GO) terms were enriched throughout the mechanical stimulus. In particular, fatty-acid ligase genes were significantly up-regulated in both the GO analysis and the gene set enrichment analysis (GSEA) throughout all analyzed time points. Meanwhile, we also identified up-regulated melanin biosynthesis pathway genes at an early time point, but they were less enriched later and were expected to produce DOPA-melanin or catechol-melanin. In summary, through the combination of de novo genome assembly and transcriptome analysis, we assembled the first pseudo-chromosome-level genome of East Asian *A.bisporus*. We also identified mechanical stimulus-responsive genes and their functional annotations which implied melanin biosynthesis genes are up-regulated to introduce browning phenotype and fatty-acid ligase genes to be up-regulated in *A. bisporus*.

## 2. Materials and Methods

### 2.1. A. bisporus Strain and DNA Isolation

*Agaricus bisporus* strain KMCC00540 was obtained from the strain collection of the Ginseng Research Division at the National Institute of Horticultural and Herbal Science (NIHHS) in South Korea. The mycelia were cultured on CDA medium (4% dried compost, 0.7% malt extract, 1% sucrose, and 2% agar) at 25 °C in the dark for 2 months. Genomic DNA was extracted using a DNeasy Tissue Kit (Qiagen, Valencia, CA, USA).

### 2.2. Genome Sequencing and Assembly

Oxford Nanopore and Illumina MiSeq libraries were prepared using a rapid sequencing kit (SQK-RAD004, Oxford Nanopore Technologies: Oxford, UK) and TruSeq Nano DNA kit (Illumina, San Diego, CA, USA), in accordance with the manufacturers’ instructions. The de novo genome assembly of ONT long reads and Illumina short reads was performed using SMARTdenovo (https://github.com/ruanjue/smartdenovo (accessed on 29 June 2022)) and a Platanus assembler (http://platanus.bio.titech.ac.jp/ (accessed on 29 June 2022)), respectively [25,26]. After assembly, the contigs were polished with Nanopolish (https://nanopolish.readthedocs.io/en/latest/ (accessed on 29 June 2022)) and Plion (https://github.com/broadinstitute/pilon/wiki (accessed on 29 June 2022)), and scaffolding was performed with Japsa v1.7-05b (https://github.com/mdcao/japsa (accessed on 29 June 2022)) [27,28]. The polished contigs of *A. bisporus* KMCC00540 were then subjected to pseudo-chromosome-level assembly based on previously reported reference genomes (*A. bisporus* H97, GCA_000300575.2 and *A. bisporus* H39, GCA_001682475.1) by Nucmer v4.0.0beta2 (http://mummer.sourceforge.net/ (accessed on 29 June 2022)) and manually re-assembled into 13 pseudo-chromosomes and 1 unassembled contig [29]. The integrity of assembled genome was analyzed by BUSCO v5.2.2 with odb9 database of *Agaricales*. The quality of the assembled genome was further analyzed with QUAST v5.1.0rc1.

### 2.3. Comparative Genomic Analysis

The genomic sequences assembled in the current study were compared with *A. bisporus* H97 genomic sequences using the NUCMER sub-module in MUMmer v4.0.0beta2 (http://mummer.sourceforge.net/ (accessed on 29 June 2022)) with a minimum match length of 2000 bp and then visualized using Circos v0.66 (http://circos.ca/ (accessed on 29 June 2022)) [29,30]. To analyze the comparative genomics of coding genes, we ran OrthoFinder v2.5.4 with whole-protein sequences as inputs [31]. A comparison of the orthogroups identified with OrthoFinder was performed manually.

### 2.4. Gene Prediction of Assembled Genome

Genes were predicted in the genome sequences using an annotation pipeline designed in Phyzen (www.phyzen.com (accessed on 29 June 2022); Seongnam, Korea). Briefly, evidence datasets were first constructed by collecting the expressed sequence tags (ESTs) and similarity information at the amino acid level with genes of related species. Based on the evidence datasets, the first gene prediction was performed using MAKER2 v2.31.8 [32]. Then, the first training data were generated using SNAP v2006-07-28 [33]. Using the first training set, the second gene prediction was performed using MAKER2, and the second set of training data were generated using AUGUSTUS v3.3.2 [34]. Next, the third gene prediction was performed using MAKER2; no evidence-supported gene sequences with annotation evidence distance (AED) score of 1 were removed to improve the annotation quality. Finally, gene sequences with AED below 1 were designated as the final protein-coding gene set predicted from the genome sequence and subjected to functional annotation.

### 2.5. RNA Extraction and mRNA-Sequencing

For the analysis of the browning-related transcriptome in the *A. bisporus*, the fruit body was stimulated using browning induction equipment (Korea Patent No. 1023581650000, 27 January 2022) developed by the mushroom department at the National Institute of Horticultural and Herbal Science (NIHHS) in South Korea. Samples were mounted on trays with 200 mm holes drilled at regular spacing (100 mm × 100 mm). The distance between the aboveground part of the fruiting body and the frame to which the silicone roller is attached was matched equally, and then the fruiting body was stimulated using the silicone roller. *A. bisporus* treated with mechanical stimulation was sampled according to the following time points: 0, 20, 60, 120 min.

For the RNA extraction, the surface of the fruit body (100 mg) was sampled, with 3 replications per time point. The samples were frozen in liquid nitrogen and ground into powder. RNA extraction was performed using an Easy-spin™ IIp Plant RNA Extraction Kit (iNtRON Biotechnology, Seongnam-Si, Korea) following the manufacturer’s instructions. The quality of the purified RNA was measured with an Agilent 2100 Bioanalyzer, following the manufacturer’s instructions (Agilent Technologies, Santa Clara, CA, USA).

Using 1 μg of the qualified RNA in each sample, poly(A) mRNA was enriched with magnetic beads with oligo (dT) and then sheared into short fragments. The cDNA was subjected to end repair and poly(A) tailing and connected with sequencing adapters using a TruSeq Stranded mRNA Sample Prep Kit (Illumina, San Diego, CA, USA). The proper cDNA fragments, purified with a BluePippin instrument (Sage Science, Beverly, MA, USA) according to the manufacturer’s instructions, were selected for further PCR amplification. The final library sizes ranged between 350 and 450 bp. Subsequently, the libraries were subjected to paired-end sequencing with a 100 bp read length using an Illumina HiSeq 2500 platform.

### 2.6. mRNA-Sequencing Analysis and Functional Annotation

Paired-end reads were cleaned using prinseq-lite version (0.20.4) with the following parameters: min_len 50; min_qual_score 5; min_qual_mean 20; derep 14; trim_qual_left 20; trim_qual_right 20 [35]. Clean paired-end reads of each sample were aligned to the potato reference sequence using Bowtie2 [36]. RSEM 1.3.0 software was used to obtain read counts and TMM-normalized TPM (trimmed mean of M value-normalized transcripts per million) values for each transcript [37]. EdgeR version 3.16.5 was used to calculate the negative binomial dispersion across conditions for differential gene expression analysis [38]. Genes were determined to be significantly differentially expressed if they showed a >4-fold change in expression, with a false discovery rate (FDR)-adjusted *p* < 0.001.

The BLAST program, with an e-value threshold of 1 × 10^−5^ against *A. bisporus* strain JB137 protein sequences, was selected from the JGI database [39,40]. Gene Ontology (GO) term was inferred from manually annotated data from the *A. bisporus* JB137 database, and enriched GO terms were determined based on EASE score from a modified Fisher’s exact test, *p* < 0.05 [40,41,42]. Enriched GO genes were further analyzed with Gene Set Enrichment Analysis (GSEA), as described in Hong et al. [43,44,45,46,47].

## 3. Results

### 3.1. De Novo Genome Assembly of Agaricus Bisporus Strain KMCC00540

The raw data of ONT sequencing were filtered, and 76,526 high-quality reads were obtained, for a total of 1,205,861,529 bp, corresponding to approximately 40× coverage of the genome. The average read length was 15,758 bp, with an N50 length of 16,545 bp. The trimmed ONT reads were assembled into contigs using SMARTdenovo with default parameters and yielded 103 contigs with N50 length of 609,681 bp. Further, 10,393,640 paired-end raw reads were generated using the MiSeq platform system and applied for polishing with Pilon and scaffolding with Japsa. Then, scaffolds were mapped and re-assembled in comparison with a previously assembled chromosome-level genome of *A. bisporus* strains H97 and H39 using NUCMER software to assemble our genome to pseudo-chromosome level (Figures S1 and S2). The genome of strain KMCC00540 was assembled into 13 chromosomes and 1 unassigned contig. The pseudo-chromosome-level genome had high integrity of 91.4% analyzed with BUSCO.

Then, we compared our genome with the assembled genomes from other strains. Among all chromosome-level genomes, our genome had the longest sequence in total and the largest contig. The N50 contig and N90 contig were the longest of every assembled genome sequence, with N50 length of 2.75 Mbp and N90 length of 1.7 Mbp. Our genome had GC content of 46.45% which is similar to other genomes. In summary, we assembled a pseudo-chromosome-level genome of the Korean strain of *A. bisporus* (Table 1).

**Figure 1:**
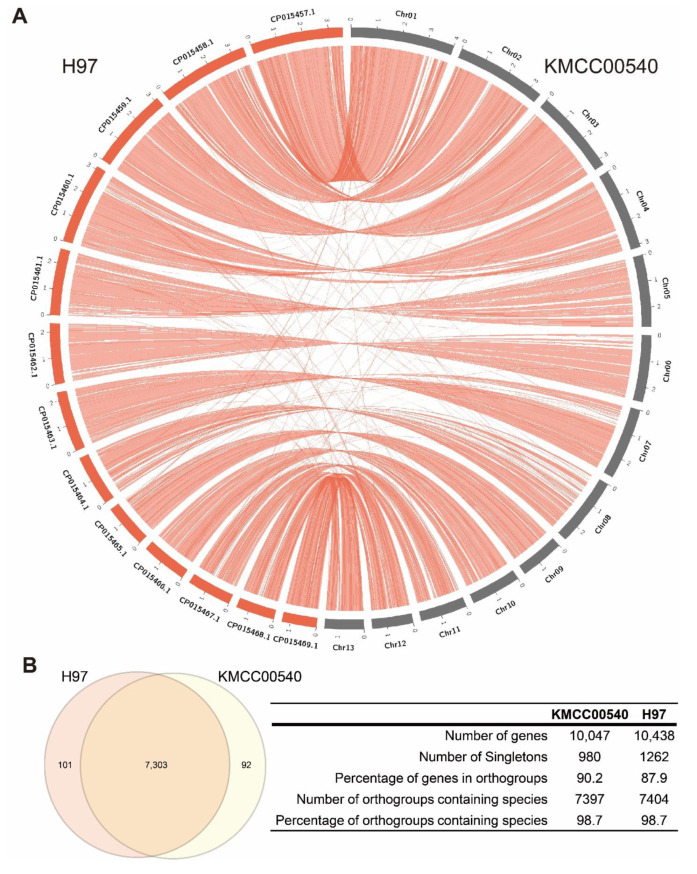
Comparative analysis of the circular diagrams and gene clustering between two *A. bisporus* genomes. (**A**) Circos plots representing the three *A. bisporus* genomes, with structural alterations noted. Inter-chromosomal and intra-chromosomal rearrangements for the two strains are shown in red. (**B**) Venn diagram of the distribution of shared gene families (orthologous clusters) in *A. bisporus* KMCC00540 and H97 strains. The numbers of each component are shown in the table.

To analyze the genomic features of the de novo assembled genome, we performed a comparative genomic analysis. The *A. bisporus* strain KMCC00540 genome had almost all the genomic blocks of *A. bisporus* strain H97, with minor rearranged genomic blocks (Figure 1A). Then, we annotated the genes and obtained 10,047 coding genes from KMCC00540. The average length of genes was 2021 bp, with mean number of exons per genes of 6.62; the mean length of the exons was 223 bp. Among those 10,047 coding genes, 600 genes coded proteins of 100 or fewer amino acids, while 127 genes coded proteins of 2000 or longer amino acids; the average length of the coded proteins was 516 amino acids. With this set of annotated coding genes, we performed ortholog search analysis with the coding genes of *A. bisporus* strain H97. Two strains had almost the same set of orthologous coding genes (7303 orthologous groups, 90.2% shared). Thus, we performed transcriptome analysis with our *A. bisporus* strain KMCC00540 as a reference genome for *A. bisporus* (Figure 1B).

### 3.2. Time-Course Transcriptome Analysis of Mechanical Stimulus Treated Button Mushroom

**Figure 2:**
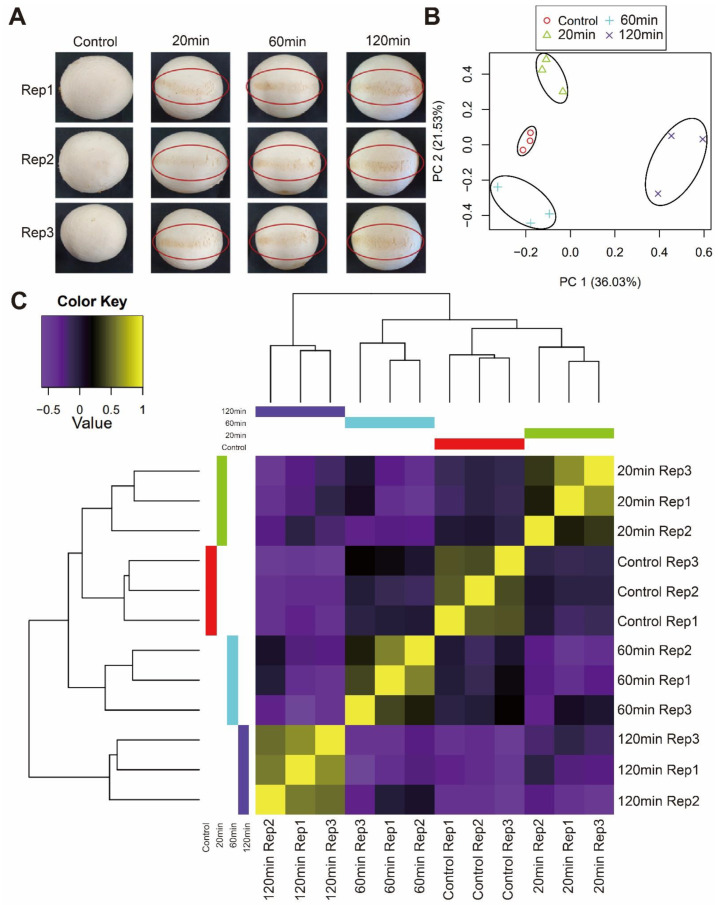
Transcriptome analysis of *A. bisporus* strain KMCC00540 with mechanical stimulus. (**A**) Phenotype of mechanical stimulus-treated *A. bisporus* after 20, 60, and 120 min. (**B**) Principal component analysis (PCA) and (**C**) hierarchical clustering analysis by time after mechanical stimulus. The repetitions of each time and control are grouped.

We identified a clear browning phenotype from a mechanical stimulus on *A. bisporus* strain KMCC00540 after 20 min and performed a time-course transcriptome with the fruit body at 0, 20, 60, 120 min after a brief mechanical stimulus (Figure 2A). After read trimming, we obtained 15,195,642 reads (2.191 Gb) before the mechanical stimulus and then 15,167,584 reads (2.193 Gb) at 20 min, 14,686,442 reads (2.153 Gb) at 60 min, and 14,385,647 reads (2.089 Gb) at 120 min. Principal component analysis (PCA) and a sample correlation matrix ensured that sample correlations among the same treatments and among different treatments were available for later transcriptome analysis (Figure 2B,C).

### 3.3. Functional Annotation of Mechanical Stimulus Treated Transcriptome in Time Course

**Figure 3:**
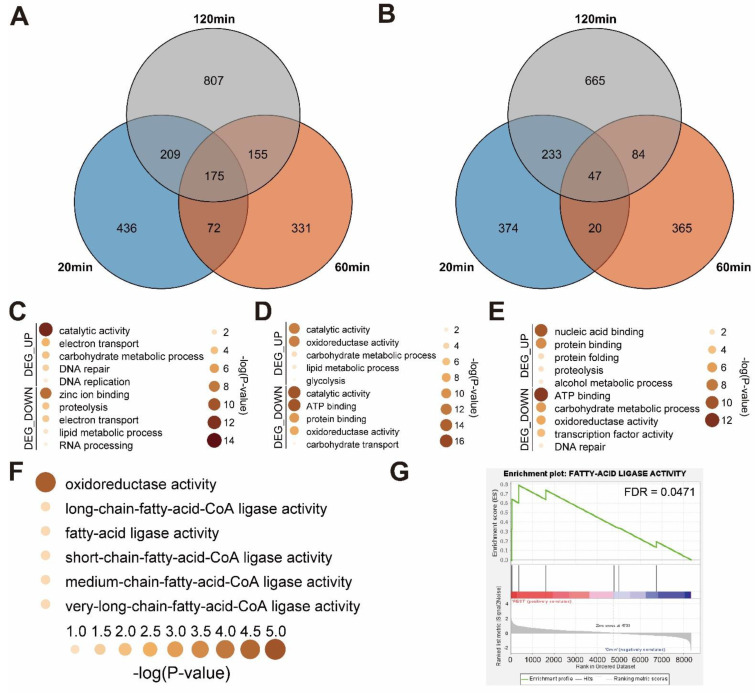
DEG analysis and functional annotation of DEGs of *A. bisporus* strain KMCC00540 under mechanical stress conditions. (**A**) Venn diagram analysis to visualize 20, 60, and 120 min dependent up-regulated DEGs. (**B**) Venn diagram analysis to visualize 20, 60, and 120 min dependent down-regulated DEGs. (**C**–**F**) GO analysis of identified DEGs. Up-regulated DEGs are indicated as DEG_UP, down-regulated DEGs are indicated as DEG_DOWN. (**C**) GO terms for DEGs at 20 min. (**D**) GO terms for DEGs at 60 min. (**E**) GO terms for DEGs at 120 min. (**F**) GO terms for common up-regulated DEGs under three conditions. (**G**) Gene-set enrichment analysis of fatty-acid ligase activity genes in mechanical stimulus treated samples compared with non-treated control.

To identify the transcriptomic changes the mechanical stimulus causes in *A. bisporus* over a time course, we identified differentially expressed genes (DEGs) at each time point by comparing them with 0 min controls (Figure 3A,B). We identified 892 up-regulated and 674 down-regulated DEGs 20 min after mechanical stimulus, 734 up-regulated and 516 down-regulated DEGs after 60 min, and 1347 up-regulated and 1030 down-regulated DEGs after 120 min (Figure 3A,B). Among these, 175 were common in up-regulated DEGs, while 47 were common in the down-regulated.

Then, we manually annotated gene ontology (GO) terms using GO annotation data of *A. bisporus* strain JB137. Among 10,047 genes, 4184 were each annotated with GO terms, which was comparable with the original annotated database. With these GO annotation data, we performed a functional annotation analysis on the DEGs we identified from the time-course mechanical stimulus-treated *A. bisporus* samples. In the 20 min samples, catabolytic activity genes and DNA replication-related genes were up-regulated on GO analysis, while proteolysis-related genes and RNA-processing genes were down-regulated (Figure 3C). In the 60 min samples, protein-folding-related genes and proteolysis genes were up-regulated, while transcription factors were down-regulated (Figure 3D). In the 120 min samples, glycolysis-related genes were up-regulated, while carbohydrate transport genes were down-regulated (Figure 3E). In summary, *A. bisporus* seemed to regulate recovery-related genes (e.g., for protein folding) and glycolysis. Interestingly, fatty-acid ligase activity genes were up-regulated in all the mechanical stimulus-treated samples, which were also confirmed to be enriched in a gene-set enrichment analysis (GSEA) (Figure 3F,G and Figrue S4). Meanwhile, the mechanically stressed up-regulated genes in *Volvariella volvacea* were not up-regulated to a notifiable level (Figure S3). In summary, *A. bisporus* regulated many genes, possibly recovery-related, while strongly up-regulating fatty-acid ligase genes in response to a mechanical stimulus (Figure 3).

### 3.4. Melanin Biosynthesis Genes Were Moderately Up-Regulated in Response to Mechanical Stimulus

**Figure 4:**
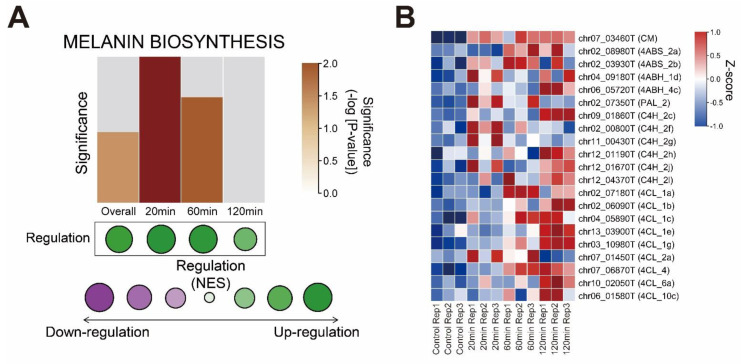
Melanin biosynthesis genes were up-regulated in response to mechanical stimulus. (**A**) Summary of gene set enrichment analysis (GSEA) of melanin biosynthesis pathway proposed in *A. bisporus.* GSEA analysis was performed in comparison with 0 min samples with indicated sample conditions. Bar graph indicates relative significance of enrichment in each condition; higher bar with deeper brown color indicates significant enrichment. Regulation patterns of melanin biosynthesis genes are indicated as colored dots. Green dots indicate up-regulation in each condition, while purple dots indicate down-regulation. Bigger dots indicate regulation was even significant in each condition. Melanin biosynthesis genes are indicated in Figure S5. (**B**) Heatmap of leading-edge genes identified in GSEA analysis. CM; chorismate mutase, 4ABS; 4-aminobenzoate synthase, 4ABH; 4-aminobenzoate hydroxylase, GGT; γ-glutaminyltransferase, PDHtase; prephenate dehydratase, PDH; prephenate dehydrogenase, AT; (4-hydroxy) phenylpyruvate aminotransferase, PPO; polyphenoloxidase (tyrosinase), PAL; phenylalanine ammonialyase, C4H; Trans-cinnamate-4-monooxygenase, 4CL; 4-coumarate CoA ligase, GHB; γ-l-glutaminyl-4-hydroxybenzene, PAP; *p*-aminophenol, DOPA; 3,4-dihydroxyphenylalanine.

**Figure 5:**
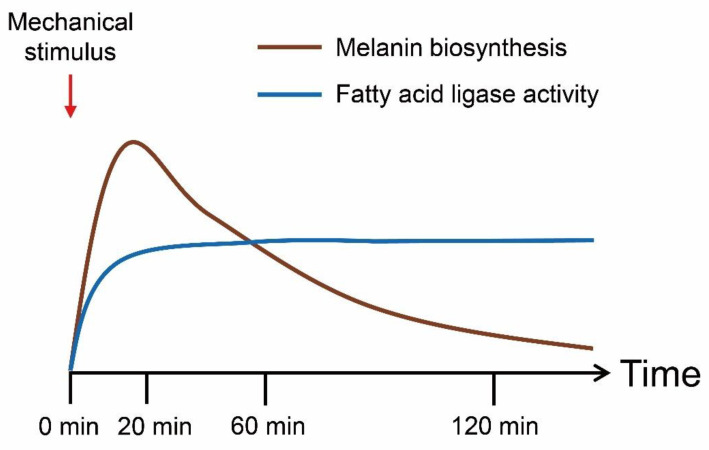
Proposed transcriptomic response after mechanical stimulus in *A. bisporus*.

To identify the mechanism of the browning phenotype in the transcriptome, we analyzed bibliographical references and summarized the melanin biosynthesis pathway in *A. bisporus* (Figure S5). Interestingly, we could not identify GGT (γ-glutaminyltransferase), which is required for PAP-melanin (*p*-aminophenol type melanin) and GHB-melanin (γ-l-glutaminyl-4-hydroxybenzene type melanin) in our genome-guided gene annotation (Figure S4). We performed a GSEA analysis of the melanin biosynthesis genes and found them moderately enriched in the mechanical stimulus-treated samples (Figure 4A). Most leading-edge genes were enriched in catechol-type melanin biosynthesis genes (Figure S5 and S6). Then, we performed a GSEA analysis of the time-course transcriptomes and identified melanin biosynthesis genes as the most up-regulated at early time points but less enriched at later times (Figure 4). In summary, we identified the mechanical stimulus-responsive genes, with their functional annotation, that explained the browning phenotype (Figure 5).

## 4. Discussion

We assembled a pseudo-chromosome-level genome of white *A. bisporus* strain KMCC00540 through a comparative assembly with the genome sequence of *A. bisporus* strains H97 and H39 (Figure 1, Figures S1 and S2). Our genome consisted of 13 chromosomes and 1 unassigned contig that was predicted to have 10,047 genes (Figure 1). White *A. bisporus* strains suffer from a browning phenotype even from slight mechanical stress. Here, we addressed how it is regulated (Figure 2). As a result, we identified 3699 DEGs from every sample (Figure 3A,B). Those genes seemed to be enriched with various GO terms, and we could clearly identify fatty-acid ligase genes as being highly up-regulated throughout the observation times (Figure 3C–G). We also identified up-regulated melanin biosynthesis genes, especially at the early time point, probably by introducing DOPA-melanin or CATECHOL-melanin in response to a mechanical stimulus (Figure 4 and Figure S6). In summary, we found white button mushrooms up-regulated with fatty-acid ligase genes and melanin biosynthesis genes at different time points, which lowers their commercial value; we also identified mechanical-stimulus-responsive genes in button mushrooms (Figure 5).

We assembled a pseudo-chromosome-level genome through a comparison among genomic sequences of our contig-level assembly with *A. bisporus* strains H39 and H97 (Figures S1, S2 and S7). Our genomic sequence had 33.03 Mbp with 13 chromosomes (32.78 Mbp) +1 unassigned contig, while the genome of strain H39 had 30.70 Mbp with 16 chromosomes and *A. bisporus* strain H97 had 30.42 Mbp with 13 chromosomes (Table 1). Interestingly, the genomes of H39 and H97 shared the most genomic block: 1 Mbp genomic blocks were absent even though they originated as a homokaryon of a single strain, Horst U1 [8]. This genomic variation originated from the life cycles of mushroom species, which have a relatively longer haploid stage and a sexual reproductive mechanism. Even with this background, our assembled KMCC00540 genome seemed to possess both *A. bisporus* H39 and H97 genomic elements with an unassigned contig of only 250 Kbp (Figure S7). Based on the breeding history of *A. bisporus*, we assembled a genome to be representative of *A. bisporus* that was a genomic sequence of a South Korean cultivar [8,11].

We could identify how mechanical stress induced browning in our transcriptome data (Figure 4). Interestingly, chorismate mutase and 4-aminobenzoate synthase were somewhat up-regulated throughout the observed time course, whereas 4-coumarate CoA ligase was up-regulated at later time points. In studies aiming to identify browning phenotype-correlated genes through combining genetic analysis and QTL, many QTLs included melanin biosynthesis pathway genes such as CM, PALs, and PPOs [46,47]. These findings confirm our explanation of how browning is regulated and further indicate that targeting these genes in molecular breeding methods such as CRISPR-Cas9 might lead to browning-insensitive types. Another approach might be utilizing antioxidant chemicals as in previous reports [4,5,6,7,16]. These chemical methods are easily anticipated by the melanin biosynthesis pathway itself, which includes many oxidative enzymes (Figure S5). In summary, our study provides not only a putative method of inhibit browning but also an explanation of browning itself in response to mechanical stimulus.

Time-course transcriptomic analysis upon mechanical stimulus clearly indicated that fatty-acid ligase genes were significantly up-regulated throughout the observed time course (Figure 3). This indicated that a mechanical stimulus introduces longer fatty-acid varieties in *A. bisporus*. A previous study on the mechanical stimulus effect on *A. bisporus* indicated weight loss and textural change; a loss in firmness was especially observed with the browning effect [15]. Meanwhile, a longer fatty-acid length is known to reduce membrane permeability [48], but this contradicted our transcriptome data in which reducing membrane permeability seemed to induce harder texture than control. Interestingly, in a previous metabolomic analysis, browning-sensitive cultivars of *A. bisporus* were significantly enriched with unsaturated fatty acids having a chain length of 16 (C16) and 18 (C18) biosynthesis varieties [24]. In combination, unsaturated forms of long-chain fatty-acids and very-long-chain fatty-acids are expected to accumulate to enhance membrane permeability since our transcriptome gave no sign of desaturase gene expression changes (Figure 3). Thus, our transcriptome also explained a previously observed phenotype coupled with browning.

The melanin biosynthesis pathway had been thought to produce four subtypes in *A. bisporus* (Figure 4A). However, at least in our genomic data, we could not annotate γ-Glutaminyltransferase (GGT) (Figure S5). This was due to a lack of reference protein sequences as indicated in a previous report; thus, it is possible that PAP-melanin and GHB-melanin are obsolete types of *A. bisporus* or that another type of enzyme might be responsible for catalyzing *p*-aminophenol into γ-l-glutaminyl-4-hydroxybenzene (Figure 4A and Figure S5). On BLASTp analysis of rat GGT5 (uniprot ID: Q9QWE9), we identified two potential GGTs, chr01_08330T and chr02_05810T, but the functional correlations of those with melanin biosynthesis is unclear (Table S1).

Our transcriptome data on the wounding of responsive genes did not correlate with findings from a previous report (Figure S3). The reason might be that the previous report treated scalpel-induced injury after incubation in a sanitizing solution and then analyzed the expression level after at least 3 days, whereas our transcriptome data concentrated on earlier time points [49]. Meanwhile, we identified three manganese peroxidase genes, *chr03_02290T*, *chr03_02740T*, and *chr02_01230T*, that were up-regulated in response to a mechanical stimulus (Figure S4). This correlated with previous reports on fungal response to injury and rapid ROS accumulation within 5 min [50]. In our transcriptome, accumulation was later than 5 min, which explains the negative feedback loop response for ROS (Figure S5). One more interesting finding from our transcriptome analysis was that in the putative transcription factor involved in melanin biosynthesis, four photoregulator B homolog genes were up-regulated at different time points. *Chr05_03540T* and *chr05_04750T* were up-regulated early, while *chr03_03430T* and *chr06_06200T* were up-regulated later (Figure S5). This suggested that *chr05_03540T* and *chr05_04750T* might regulate an early response in a mechanical stimulus-treated condition, while *chr03_03430T* and *chr06_06200T* might regulate late responses. Early response included both melanin biosynthesis and fatty-acid ligase activity, while late response included only fatty-acid ligase activity (Figure S4 and Figure 5). In short, our report demonstrates how mechanical stimuli, including the accumulation of melanin, reduce the commercial value of white *A. bisporus*.

## 5. Conclusions

The edible button mushroom *Agaricus bisporus* is popular worldwide, but the lack of comprehensive genomic sequence data hinders molecular analysis and breeding efforts. We assembled an *A. bisporus* (strain KMCC00540) genome sequence and scaffolded to pseudo-chromosomal level through a comparative genomic pipeline. On the de novo assembly of KMCC00540 and comparative genomic analysis with the public chromosomal-level genome of *A. bisporus* strain H97, we found that our genome can represent *A. bisporus* globally. Then, we performed time-course transcriptome analysis to identify how mechanical stimuli, including melanin accumulation, lower the commercial value of white varieties of *A. bisporus*. In the analysis, we identified not only up-regulated melanin biosynthesis genes early after mechanical stimulus but also highly up-regulated fatty-acid ligase genes throughout our observation. In combination with previous findings on mechanical stress inducing the biosynthesis of unsaturated fatty-acid varieties, we determined that unsaturated/long-chain fatty-acids might be responsible for reduced firmness and accelerated weight loss in response to a mechanical stimulus. Finally, we identified a transcription factor that might govern the post-harvest transcriptomic response, photoregulator B. In conclusion, our comprehensive in silico analysis well explained how a mechanical stimulus affects the post-harvest transcriptome in *A. bisporus* and correlates with a phenotype, thus, providing insight into how browning is regulated as well as putative insights into firmness regulation with a mechanical stimulus.

## Figures and Tables

**Table 1:** Genome information comparison of *A. bisporus* strains.

Strain	KMCC00540	H97	H39	AB58	H119	ARP23	**JB137-s8**
Assembly level	Chromosome	Chromosome	Chromosome	Chromosome	Chromosome	Scaffold	Contig
Number of contigs (≥0 bp)	14	13	16	18	16	169	2016
Number of contigs (≥10,000 bp)	14	13	16	18	16	167	121
Number of contigs (≥50,000 bp)	14	13	16	17	16	121	52
Total length (≥0 bp)	33,281,038	30,417,844	30,702,502	30,069,126	30,702,502	33,487,476	32,614,392
Number of contigs	14	13	16	18	16	169	2015
Largest contig (bp)	3,927,320	3,550,205	3,464,045	3,600,717	3,464,045	1,506,893	2,973,556
Total length (bp)	33,281,038	30,417,844	30,702,502	30,069,126	30,702,502	33,487,476	32,613,893
GC (%)	46.45	46.5	46.64	46.53	46.64	46.34	46.59
N50	2,750,289	2,550,681	1,931,181	2,300,414	1,931,181	350,711	1,225,131
N90	1,704,996	1,688,379	1,323,473	1,336,791	1,323,473	91,735	19,667

## Data Availability

The complete genome sequence of *A. bisporus* KMCC00540 has been deposited in NCBI under the GenBank numbers CP039873–CP039885. All the raw sequence data described in this paper are available through NCBI’s BioProject (PRJNA534383) and BioSample (SAMN11489404) database.

## Supplementary Materials

The following supporting information can be downloaded at: https://www.mdpi.com/article/10.3390/jof8080886/s1. Figure S1: Chromosome-level rearrangement and synteny analysis between *A. bisporus* cultivar KMCC00540 and H97. Blue bars indicate same orientation synteny, and red bars indicate reverse orientation synteny for at least 2000 bp blocks. Each white bar indicates chromosomes, and grey parts indicate matched genomic blocks; Figure S2: Chromosome-level rearrangement and synteny analysis between *A. bisporus* cultivars KMCC00540 and H39. Blue bars indicate same orientation synteny, and red bars indicate reverse orientation synteny at least for 2000 bp blocks. Each white bar indicates chromosomes, and grey parts indicate matched genomic blocks; Figure S3: Expression heatmap of previously reported mechanical injury-responsive genes. Z-score values were visualized throughout our transcriptome samples; Figure S4: GO analysis of identified GO term DEGs. (A) GO terms of up-regulated DEG from 20 min. (B) GO terms of up-regulated DEG from 60 min. (C) GO terms of up-regulated DEG from 120 min. (D) GO terms of common up-regulated DEGs of three conditions. (E) GO terms of down-regulated DEG from 20 min. (F) GO terms of down-regulated DEG from 60 min. (G) GO terms of down-regulated DEGs from 120 min; Figure S5: Melanin biosynthetic pathway and expression levels of each steps. Several enzymes are shared by subset of reactions. Z-score values are visualized. Sample coordination is visualized under 4CL genes; Figure S6: GSEA analysis on Melanin biosynthetic pathway. (A) GSEA analysis in comparison of 0min and REST. (B) GSEA analysis in comparison of 0min and 20 min. (C) GSEA analysis in comparison of 0min and 60min. (D) GSEA analysis in comparison of 0min and 120min; Figure S7: Comparative genomic analysis among three cultivars. Circos plots representing the three *A. bisporus* genomes, with structural alterations noted. Inter-chromosomal rearrangements for the three strains are shown in red, blue and grey. Red colored line indicates synteny between H97 and KMCC00540, blue colored line indicates synteny between H39 and KMCC00540, and grey colored line indicates synteny between H39 and H97; Table S1: Time-course transcriptome data on the mechanical stimulus-treated *A. bisporus* strain KMCC00540.
