# Supplementary material for: Pseudo-Chromosomal Genome Assembly in Combination with Comprehensive Transcriptome Analysis in Agaricus bisporus Strain KMCC00540 Reveals Mechanical Stimulus Responsive Genes Associated with Browning Effect
Source: J Fungi (Basel). 2022 Aug 22;8(8):886. doi: 10.3390/jof8080886 (PMC9410529; doi:10.3390/jof8080886)
Supplement: Supplementary file 1 [file jof-08-00886-s001.zip › Supplementary_Figure_.pdf]

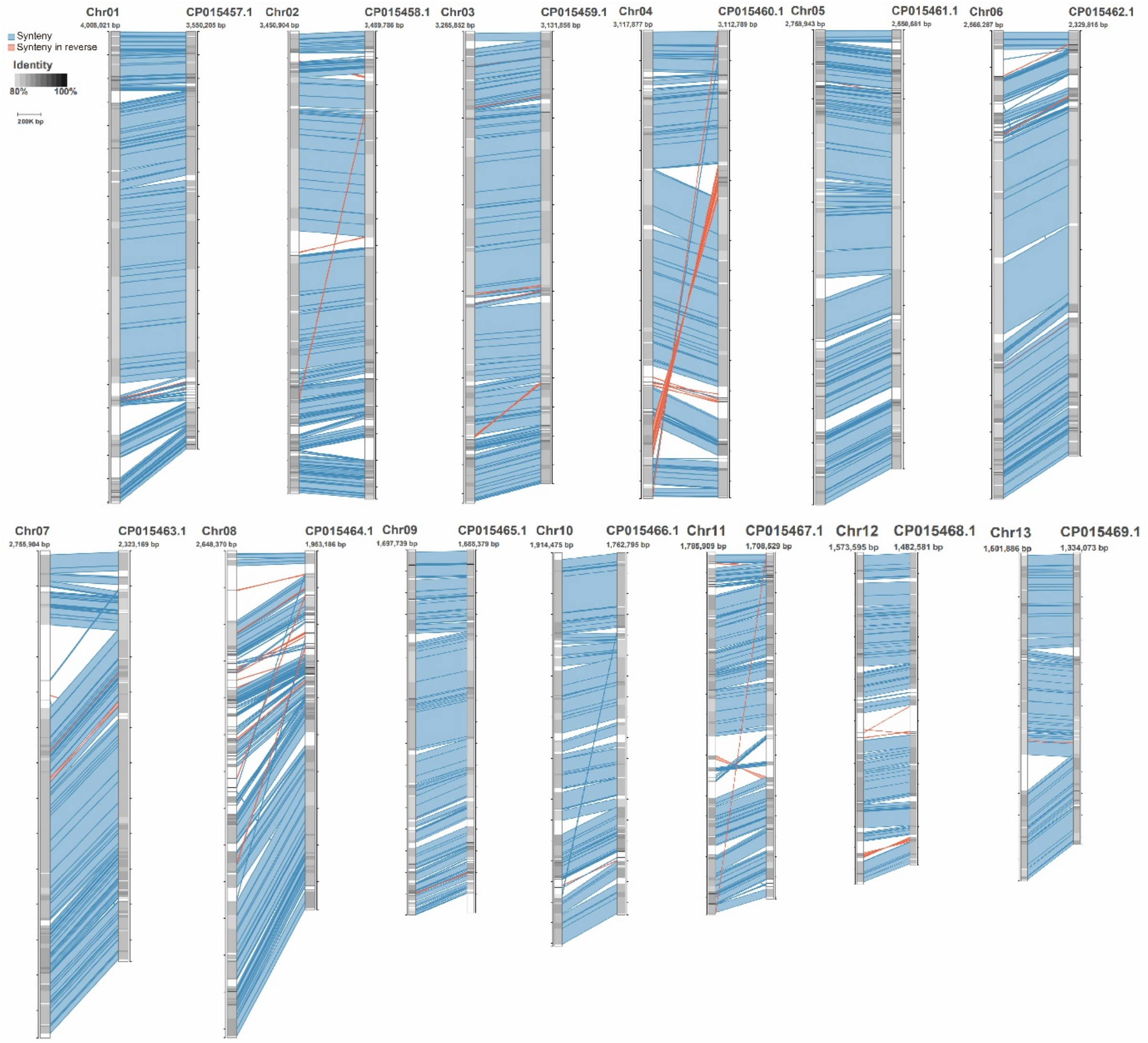

Figure S1. Chromosome level re-arrangement and synteny analysis between *A.bisporus* cultivar KMCC00540 and H97. Blue bars indicate same orientation synteny and red bars indicate reverse orientation synteny at least for 2000 bp block level. Each white bar indicates chromosomes and grey parts indicate matched genomic blocks.

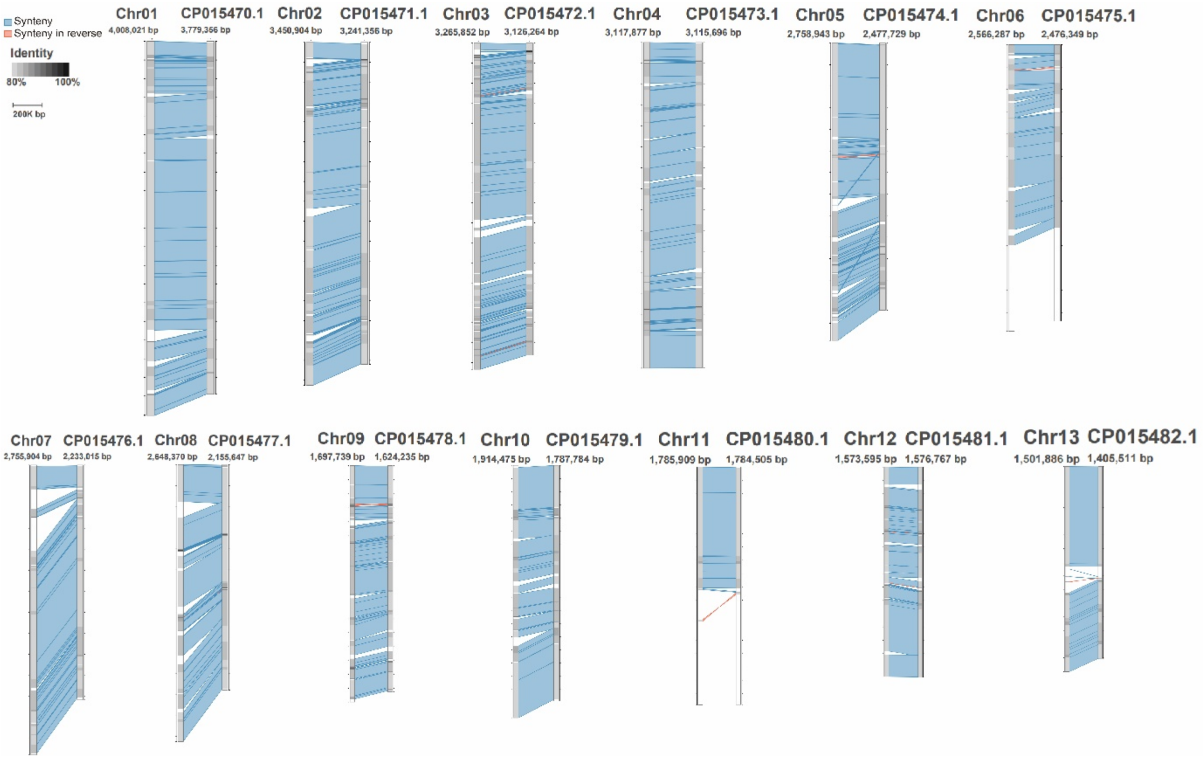

Figure S2. Chromosome level re-arrangement and synteny analysis between *A.bisporus* cultivar KMCC00540 and H39. Blue bars indicate same orientation synteny and red bars indicate reverse orientation synteny at least for 2,000 bp block level. Each white bar indicates chromosomes and grey parts indicate matched genomic blocks.

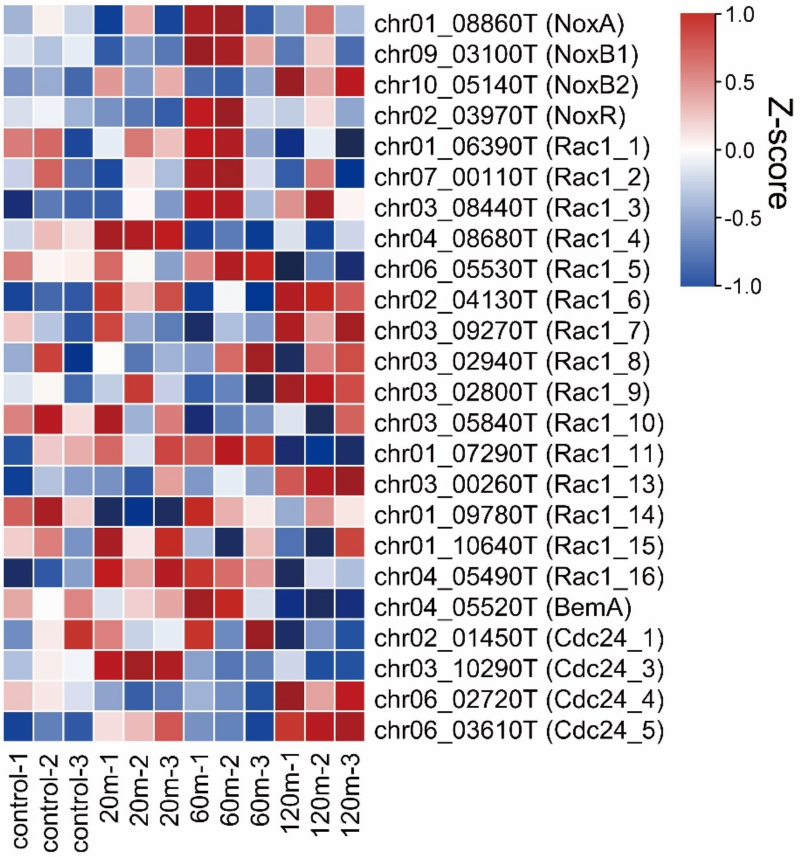

Figure S3. Expression heatmap of previously reported mechanical injury responsive genes. Z-score values were visualized throughout our transcriptome samples.

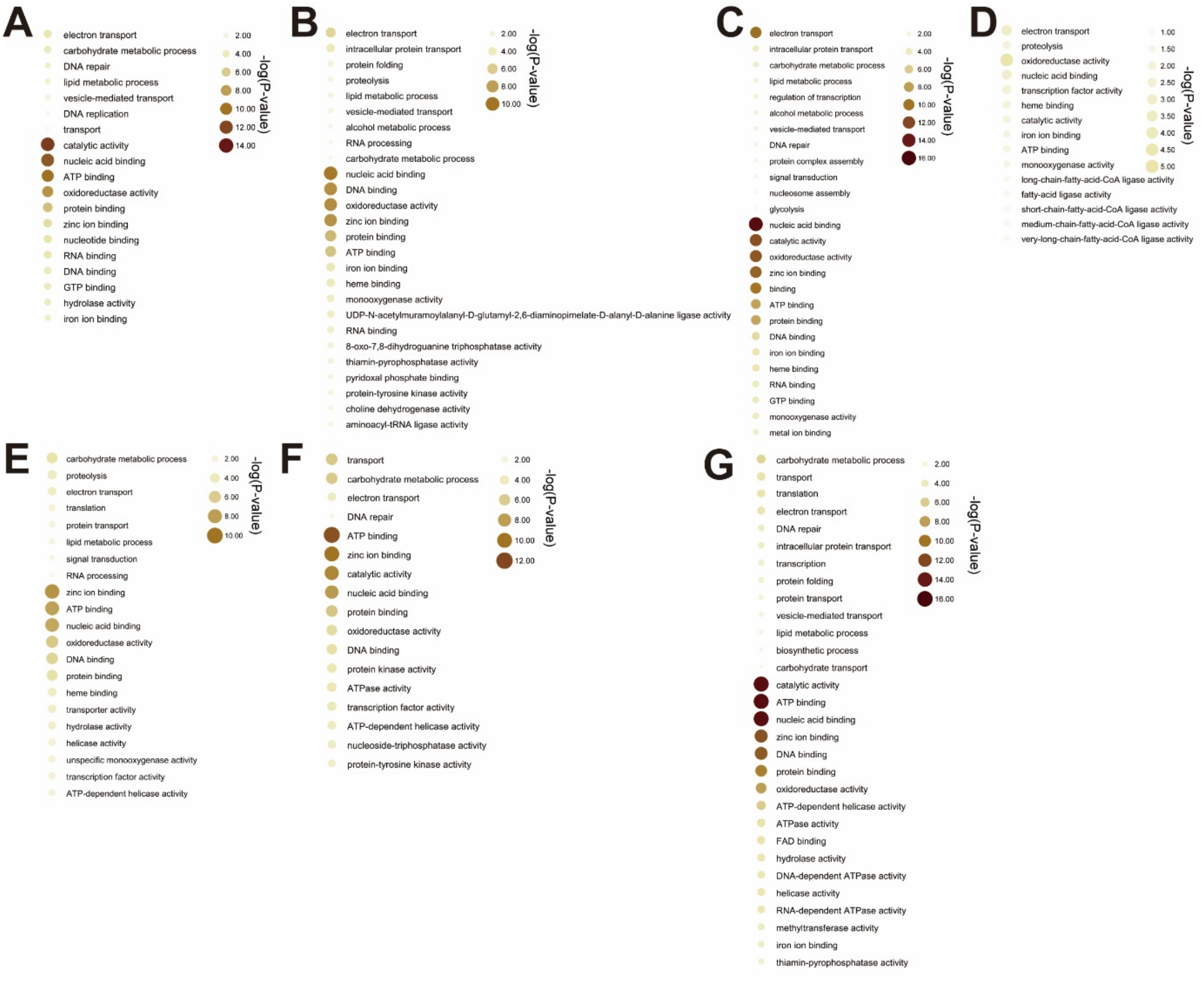

Figure S4. GO analysis of identified GO term DEGs. (A) GO terms of up-regulated DEG from 20 min. (B) GO terms of up-regulated DEG from 60 min. (C) GO terms of up-regulated DEG from 120 min. (D) GO terms of common up-regulated DEGs of three conditions. (E) GO terms of down-regulated DEG from 20 min. (F) GO terms of down-regulated DEG from 60 min. (G) GO terms of down-regulated DEGs from 120 min.

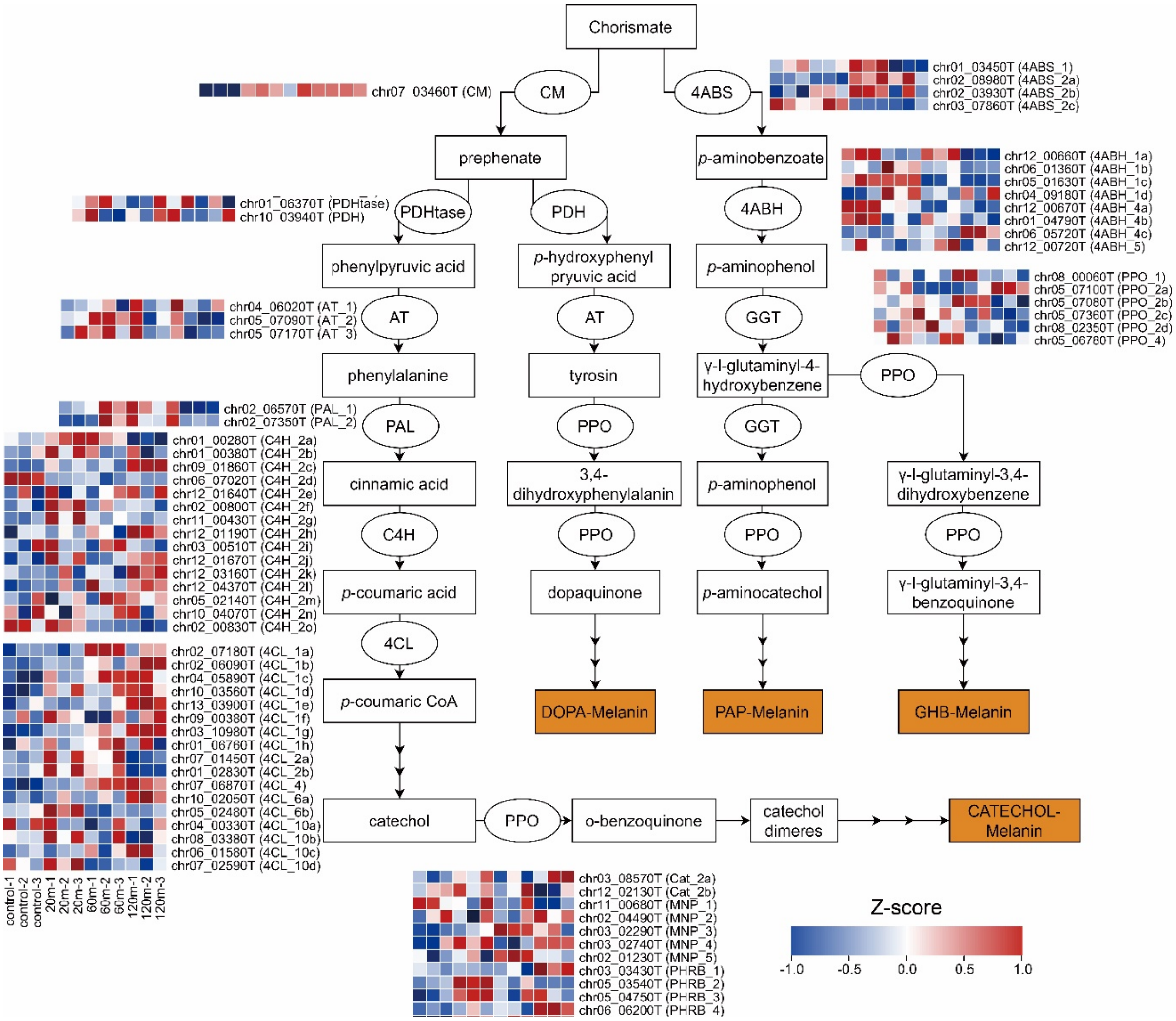

Figure S5. Melanin biosynthetic pathway and expression levels of each steps. Several enzymes are shared by subset of reactions. Z-score values are visualized. Sample coordination is visualized under 4CL genes.

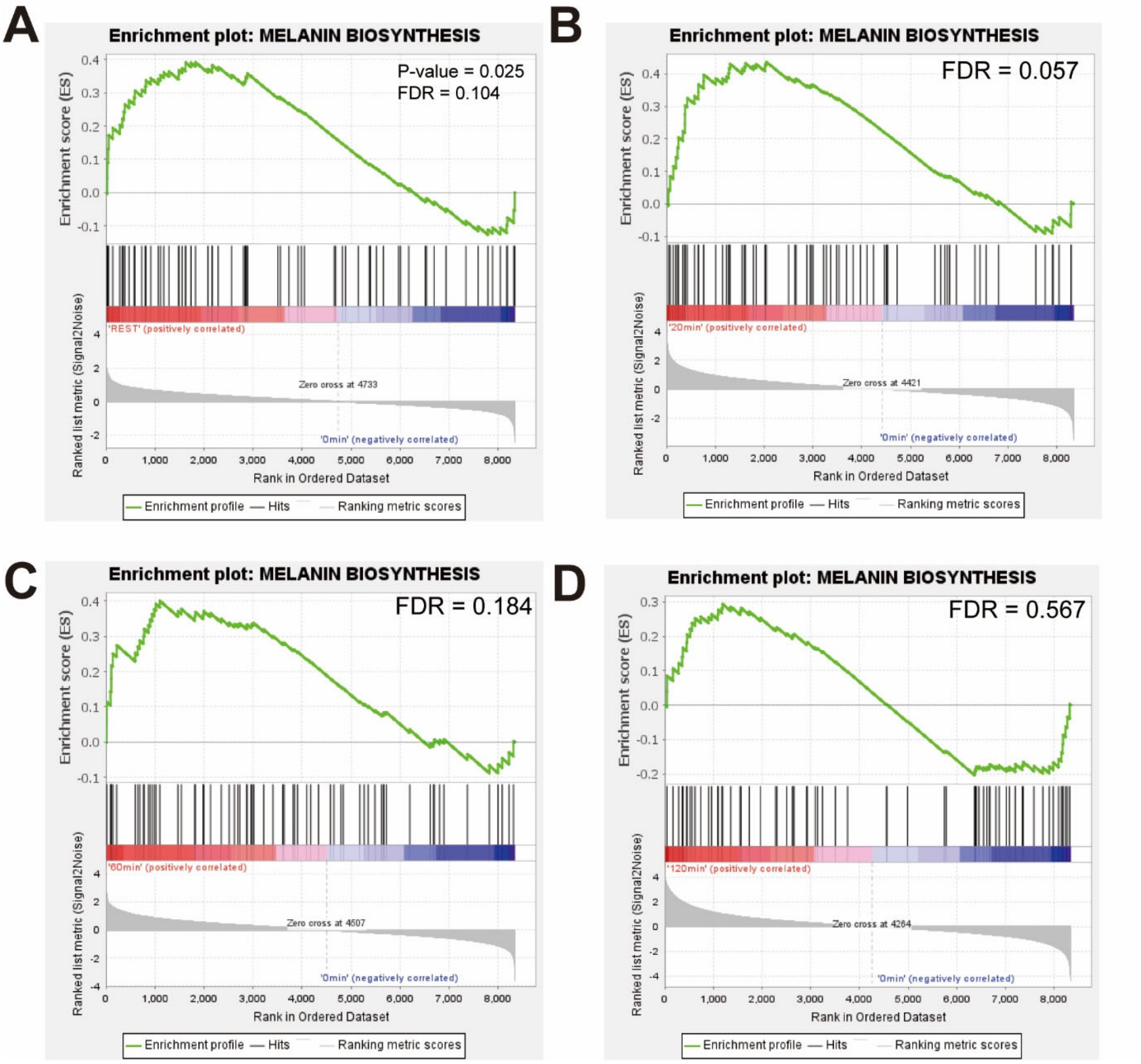

Figure S6. GSEA analysis on Melanin biosynthetic pathway. (A) GSEA analysis in comparison of 0min and REST. (B) GSEA analysis in comparison of 0min and 20 min. (C) GSEA analysis in comparison of 0min and 60min. (D) GSEA analysis in comparison of 0min and 120min.

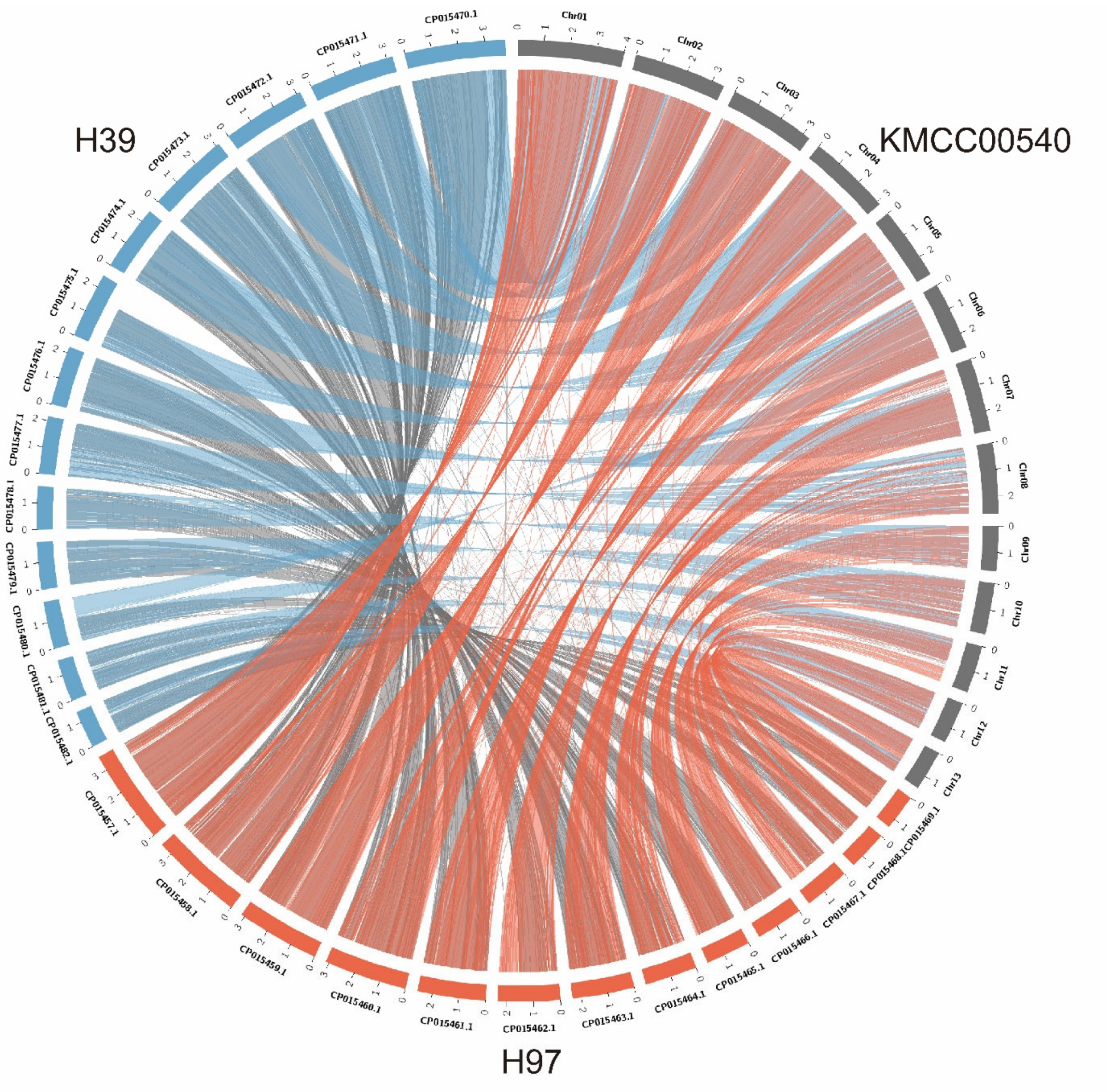

Figure S7. Comparative genomic analysis among three cultivars. Circos plots representing the three *A. bisporus* genomes, with structural alterations noted. Inter-chromosomal rearrangements for the three strains are shown in red, blue and grey. Red colored line indicates synteny between H97 and KMCC00540, blue colored line indicates synteny between H39 and KMCC00540, and grey colored line indicates synteny between H39 and H97.
